# Evidence that Moved Psychedelic Medicine from the Fringe to the Mainstream in 2022

**DOI:** 10.1192/j.eurpsy.2023.42

**Published:** 2023-07-19

**Authors:** M. Bogenschutz

**Affiliations:** Psychiatry, NYU Grossman School of Medicine, New York, United States

## Abstract

**Abstract:**

Interest in possible clinical uses for psychedelic drugs has grown steadily over the past decade. Although impressive findings from small studies stimulated considerable speculation and provided a strong justification for further study of psychedelic treatments, until very recently there was a dearth of high-quality evidence for their efficacy, mechanisms of action, and appropriate treatment models for clinical use. However, during the past 2-3 years, there have been dramatic advances in the field. This presentation will focus on 5 publications in the field of psychedelic medicine that exemplify three important aspects of the recent progress in psychedelic research. (1) There has been a rapid increase in the number and size of controlled clinical trials of various psychedelic treatments. (2) Conceptual models for studying and potentially understanding the therapeutic effect of psychedelics have increased in sophistication and comprehensiveness. And (3) progress has been made toward developing models of treatment that would facilitate access to safe and effective psychedelic treatments, if and when they are approved by regulatory bodies. Although progress has been rapid, the field of psychedelic medicine is still in its infancy. Much more work on these and many other fronts will be necessary to discover what the study of psychedelics can contribute to healthcare and neuroscience.

**Disclosure of Interest:**

None Declared

